# Rivastigmine–Benzimidazole Hybrids as Promising Multitarget Metal-Modulating Compounds for Potential Treatment of Neurodegenerative Diseases

**DOI:** 10.3390/ijms24098312

**Published:** 2023-05-05

**Authors:** David Vicente-Zurdo, Leonardo Brunetti, Luca Piemontese, Beatriz Guedes, Sandra M. Cardoso, Daniel Chavarria, Fernanda Borges, Yolanda Madrid, Sílvia Chaves, M. Amélia Santos

**Affiliations:** 1 Centro de Química Estrutural, Departamento de Engenharia Química, Institute of Molecular Sciences, Instituto Superior Técnico, Universidade de Lisboa, Av. Rovisco Pais 1, 1049-001 Lisboa, Portugal; 2 Department of Analytical Chemistry, Faculty of Chemistry, Complutense University of Madrid, Avenida Complutense s/n, 28040 Madrid, Spain; 3 Department of Pharmacy-Pharmaceutical Sciences, University of Bari Aldo Moro, via E. Orabona 4, 70125 Bari, Italy; 4 CNC—Center for Neuroscience and Cell Biology, University of Coimbra, 3000-370 Coimbra, Portugal; 5 FMUC—Faculty of Medicine, University of Coimbra, 3000-370 Coimbra, Portugal; 6 CIQUP-IMS, Department of Chemistry and Biochemistry, Faculty of Sciences, University of Porto, Rua do Campo Alegre s/n, 4169-007 Porto, Portugal

**Keywords:** Alzheimer’s disease, Parkinson’s disease, rivastigmine hybrids, metal chelation, cholinesterases, monoamine oxidase, amyloid-β aggregation

## Abstract

With the goal of combating the multi-faceted Alzheimer’s disease (AD), a series of Rivastigmine-Benzimidazole (RIV–BIM) hybrids was recently reported by us as multitarget-directed ligands, thanks to their capacity to tackle important hallmarks of AD. In particular, they exhibited antioxidant activity, acted as cholinesterase inhibitors, and inhibited amyloid-*β* (A*β*) aggregation. Herein, we moved forward in this project, studying their ability to chelate redox-active biometal ions, Cu(II) and Fe(III), with widely recognized roles in the generation of oxidative reactive species and in protein misfolding and aggregation in both AD and Parkinson’s disease (PD). Although Cu(II) chelation showed higher efficiency for the positional isomers of series **5** than those of series **4** of the hybrids, the A*β*-aggregation inhibition appears more dependent on their capacity for fibril intercalation than on copper chelation. Since monoamine oxidases (MAOs) are also important targets for the treatment of AD and PD, the capacity of these hybrids to inhibit MAO-A and MAO-B was evaluated, and they showed higher activity and selectivity for MAO-A. The rationalization of the experimental evaluations (metal chelation and MAO inhibition) was supported by computational molecular modeling studies. Finally, some compounds showed also neuroprotective effects in human neuroblastoma (SH-SY5Y cells) upon treatment with 1-methyl-4-phenylpyridinium (MPP^+^), a neurotoxic metabolite of a Parkinsonian-inducing agent.

## 1. Introduction

The incidence of neurodegenerative diseases has been growing enormously worldwide, following the aging of the global population. Alzheimer’s disease (AD) is the most prevalent form of dementia (ca. 70%, with the total number expected to reach 100 million in 2050) [1], while Parkinson´s disease (PD) is the second most common neurodegenerative disorder, though the number of PD patients is expected to surpass 12 million by 2040 [2]. Despite the tremendous research efforts to find effective drugs for these complex neurodegenerative diseases, the main cause of their pathogenesis remains elusive, although their multifactorial nature is recognized, and several iconic hallmarks have been identified. Age is the most relevant risk factor, while the interplay of genetic factors is also believed to be important. Unfortunately, so far, there are no disease-modifying therapies able to slow or stop the ongoing neurodegenerative process, especially in AD [1]. 

Currently, the main class of approved drugs for these two neurodegenerative disorders can only compensate for the loss of specific neurotransmitters, providing symptomatic relief for a short time period. For instance, treatments approved for AD primarily compensate for the loss of cholinergic neurons and include the use of inhibitors of cholinesterases (ChE), such as acetylcholinesterase (AChE) and butyrylcholinesterase (BChE). In fact, among the six drugs approved by the US Food and Drug Administration (FDA) to treat AD, four (tacrine, donepezil, rivastigmine, and galantamine) are AChE inhibitors [3,4]. Available therapeutic strategies for PD include the administration of the dopamine precursor (L-DOPA) [5] and the use of monoamineoxidases (MAOs) inhibitors. MAOs are a family of enzymes that oxidatively deaminates monoamines such as dopamine and serotonin, with concomitant generation of H_2_O_2_. Therefore, increased MAO activity and expression are associated with the dysregulation of dopaminergic and serotonergic neurotransmission, with the associated generation of H_2_O_2_ and increased oxidative stress, another important pathological mechanism observed in AD and PD [6,7].

One of the most widely recognized main obstacles to the discovery of effective drugs for these neurodegenerative diseases has been attributed to their multifactorial etiologies. Although the pathological mechanisms of AD and PD are different, they are both characterized by proteopathy, specifically associated with protein misfolding and the formation of aggregates, which accumulate in the brain. AD is characterized by the deposition of senile plaques (the aggregation of amyloid-*β* peptide (A*β*)) and neurofibrillary tangles (linked to hyperphosphorylation of tau protein), while PD is associated with misfolded aggregates of α-synuclein (*α-Syn*) that accumulate in Lewy bodies [8]. 

Metal ions, such as iron, copper, and zinc, have also been implicated in these neurodegenerative diseases, as dysregulation of metal ions (e.g., iron, copper, and zinc) has been found in susceptible regions of patient brains [9,10]. In particular, the redox-active metal ions (Cu(I/II) and Fe(II/III)) can generate reactive oxygen species (ROS), through the Fenton reaction. Moreover, these metal ions can bind to amyloidogenic peptides and, when interlinked with other metal ions such as the non-redox-active Zn(II), they are able to modify and promote their aggregation processes [11]. 

In addition to the aforementioned etiologies, many other pathological features are shared in neurodegenerative disorders. Thus, the multifactorial nature of these illnesses has challenged the scientific community for the requirement of multi-target therapeutic agents (also called multitarget-directed ligands, MTDLs) to simultaneously tackle several pathological features. With this aim, many researchers have recently pursued this drug development strategy, which, in many cases, has involved the derivatization of natural or synthetic privileged scaffolds with relevant pharmacological activities, including already approved ChE inhibitors, as potential anti-AD and anti-PD drugs [12,13,14,15,16,17,18].

**Figure 1:**
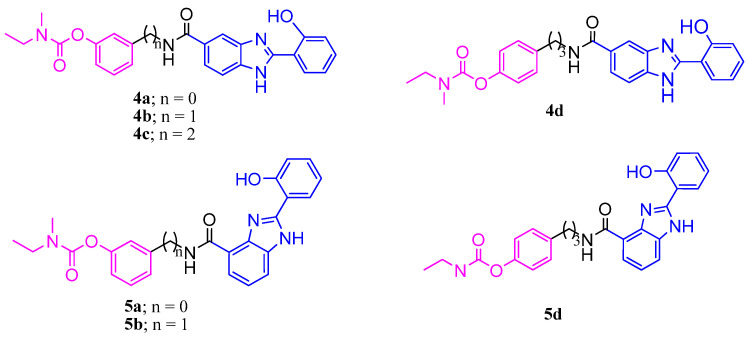
Chemical structures of the RIV–BIM hybrids.

Following a multitarget drug discovery strategy based on the drug repositioning approach (derivatization of old anti-AD drugs), we recently developed and studied two series of hybrid compounds by conjugating two AChE inhibitors (tacrine and donepezil pharmacophore) with the hydroxyphenylbenzimidazole (BIM) moiety as potential drug candidates for AD. The rationale for the introduction of the BIM unit in the developed hybrids is to provide further capacity for hitting different targets such as the inhibition of A*β* aggregation, antioxidant activity, and metal chelation, which was in fact obtained in our previous studies [19,20]. Moreover, taking into account the recognized interest in inhibiting both ChEs, more recently, we developed a novel series of molecular hybrids (RIV–BIM) derived from the conjugation of the pharmacophore of the drug rivastigmine (RIV) with the same BIM moiety as potential multitarget drug candidates for AD (see Figure 1) [21]. Thus, in addition to the well-calculated pharmacokinetic parameters we reported, these hybrids showed good inhibitory capacity of ChEs (AChE and BChE) due to the RIV moiety. On the other hand, the presence of the BIM moiety may be primarily responsible for the inhibition of A*β* aggregation, antioxidant activity, and protection against the toxicity induced by A*β* and ROS in neuroblastoma SH-SY5Y cells [21]. Notably, the inhibition of A*β* aggregation by these hybrids showed some dependence on the co-presence of Cu(II), attributable either to the possible formation of copper complexes with the bidentate (*N*,*O*) BIM moiety or to their different ability to intercalate between fibrils. Therefore, based on these encouraging results obtained for the RIV–BIM hybrids as potential anti-AD drug candidates [21] and on a recent report on a rivastigmine hybrid (MT-031) endowed with ChE and MAO inhibitory activities, as a potential neuroprotective/neurorestorative anti-PD drug [18], we decided to go further with the investigation of RIV–BIM hybrids and aimed to rationalize our previously obtained results to obtain some further insight into their potential capacity as anti-PD drugs. In fact, the disruption of cholinergic transmission is also associated with PD dementia (PDD), in addition to the existence of other commonalities and onsets between AD and PD [22].

Herein, we report a complementary study on the physico-chemical and biological properties of this set of RIV–BIM hybrids (see Figure 1). Considering the recognized interest in including metal-chelating moieties in multitarget anti-neurodegenerative drugs [12,23,24], the complexation capacity of two representative positional isomeric hybrids toward the redox-active metal ions (Cu(II) and Fe(III)) was evaluated, involving solution equilibrium studies and molecular simulations. Furthermore, since MAO inhibition has been used as a recent promising multi-target approach to treat AD and PD [17,25], the ability of these RIV–BIM hybrids to inhibit both MAO isoforms (MAO-A and MAO-B) was also evaluated and complemented with docking simulations to provide some insight into the ligand–enzyme interaction. Finally, the neuroprotective effects of RIV–BIM compounds against 1-methyl-4-phenylpyridinium (MPP^+^)-induced damage in human neuroblastoma (SH-SY5Y) cells with MPP^+^, as in vitro cell model of dopaminergic neurons death in PD, was assayed. 

## 2. Results and Discussion

### 2.1. Metal Complexation Studies

The dyshomeostasis of brain metal ions (e.g., Fe(III), Cu(II), and Zn(II)) is associated with several pathological effects of AD progression, such as A*β* accumulation, *tau* hyperphosphorylation, oxidative stress, and neuroinflammation [26,27,28]. Therefore, the use of multi-target anti-AD compounds with metal-chelating ability could play an important role in the fight against this disease [29]. The RIV–BIM hybrids contain a BIM moiety, which may act as a biometal chelator and also contribute to the dual inhibition of AChE and the inhibition of self- and Cu(II)-mediated A*β* aggregation [19,20,30]. This bidentate moiety, with (*N*,*O*) coordination mode, was already inserted by our team into hybrids containing tacrine (TAC-BIM) [19] and donepezil moieties (DNP-BIM) [30], and they showed good complexation capacity towards hard-soft metal ions. Herein, two selected RIV–BIM isomeric positional hybrids (compounds **4c** and **5a**) were studied in terms of the chelating ability for Fe(III) and Cu(II).

#### 2.1.1. Solution Equilibria

##### Acid-Base Properties

To formulate the model for the metal chelation mechanism of compounds **4c** and **5a**, their protonation constants were first determined by spectrophotometric titrations in a 50% (*w*/*w*) DMSO/H_2_O medium by fitting the experimental data with the program Psequad [31]. This working medium was adopted to perform all the equilibrium studies due to certain solubility problems of the compounds. Since the concentration of the compound used in cell studies is low (<7 μM), the final corresponding concentration of DMSO in culture media would be much lower (<1%) and also is not expected to induce modifications of biological tissues.

**Figure 2:**
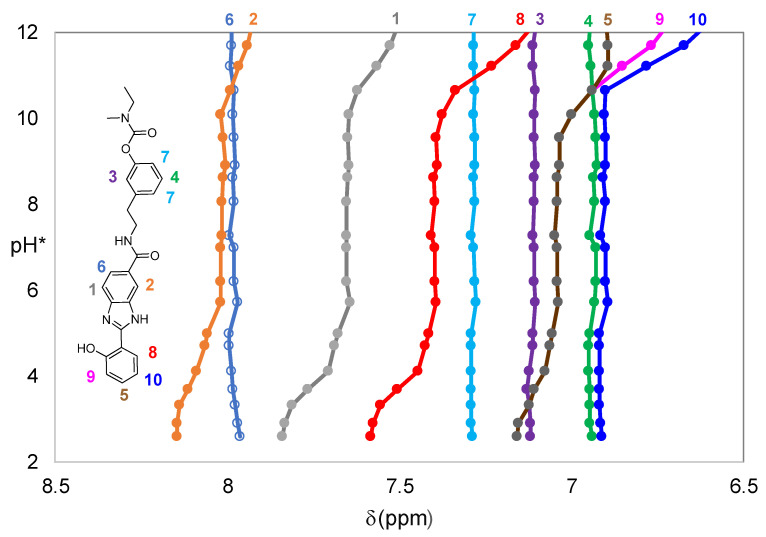
Representative ^1^H NMR titration curves of **4c** in 75% *d*_6_-DMSO/D_2_O medium (*C*_L_ = 4.5 mM) for 6.6 < δ (ppm) < 8.2; pH* value = reading value of the pH meter previously calibrated with standard aqueous buffers pH 4 and 7. Each color and number represent a specific proton of the reported molecule.

Analysis of Table 1 shows two calculated values for the protonation constants of both compounds, which may be associated with basic centers of the BIM moiety, the first one corresponding to the phenolic oxygen and the second one to the imidazole nitrogen N(3). The values obtained are in accordance with those previously determined for DNP-BIM compounds or TAC-BIM [30,32]. In fact, the ^1^H NMR titration curves of compound **4c** also evidence that, with the decrease in pH* values, the first center to be protonated is the phenolic oxygen, since downfield shifts of the resonance peaks corresponding to protons 1, 2, 8, 5, 9, and 10 can be observed in Figure 2 for a pH* range of 9.5–12. Furthermore, the more evident downfield shifts of peaks 1, 2, and 8 in the pH* range of 2.9–4.5 are in agreement with the protonation of the imidazole nitrogen N(3) of the BIM moiety. 

**Figure 3:**
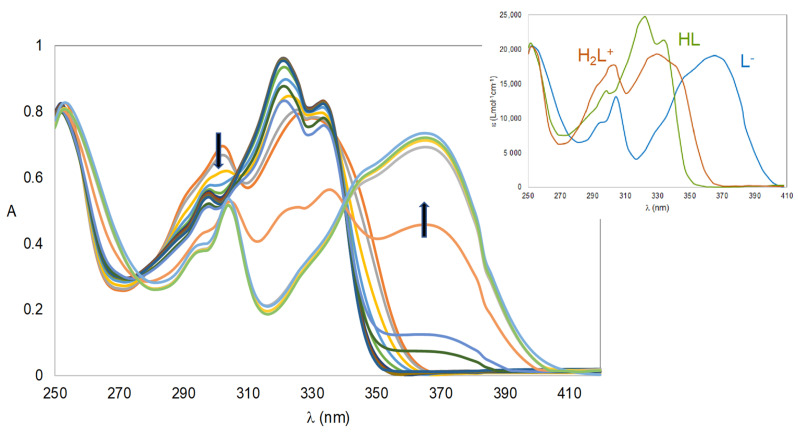
Absorbance spectra of **4c** (2.75 < pH < 11.3, *C*_L_ = 4 × 10^−5^ M) in 50% (*w*/*w*) DMSO/H_2_O and outset with individual calculated spectra by Psequad [30]. The arrows indicate the evolution of the spectra when pH increases. Each line corresponds to an absorbance spectrum obtained at a specific pH.

UV absorbance titration curves for **4c** in the pH range of 2.75−11.3 are illustrated in Figure 3. The outset of this figure shows that the fully protonated species (H_2_L^+^) of compound **4c** has three absorbance maxima at 252, 304, and 330 nm, with the maximum at 252 nm also corresponding to the species HL and L^−^. Moreover, the neutral HL species has a strong maximum at 322 nm while the fully deprotonated species L^−^ shows a strong absorption band at 366 nm. At pH 7.4 and for *C*_L_ = 1 × 10^−5^ M, to meet the physiological conditions, the predominant species for **4c** and **5a** is HL, at 97.4% and 94.9%, respectively. Although the lipo-hydrophilic character is not only determined by the charge but also by the capacity to establish solute–solvent interactions, the existence of the neutral HL species in high concentrations for both compounds explains the need to choose a 50% DMSO/water medium to perform the solution studies.

##### Metal Chelation

The metal complexation studies were performed by spectrophotometric titration, in the same 50% *w*/*w* DMSO/water medium used for both ligands alone, with fitting analysis of the UV-Vis spectra by Psequad [31]. The calculated metal-chelating equilibrium models, included in Table 1, show good chelating capacity towards Cu(II) (pCu~10.3–13.6) and Fe(III) (pFe~16.8–17.4), involving complex species with a 1:1 to 1:2 or 1:1 to 1:3 M/L ratio, respectively. Metal coordination is expected to occur primarily through the phenolic oxygen and the imidazole nitrogen N(3) of the BIM moiety.

**Figure 4:**
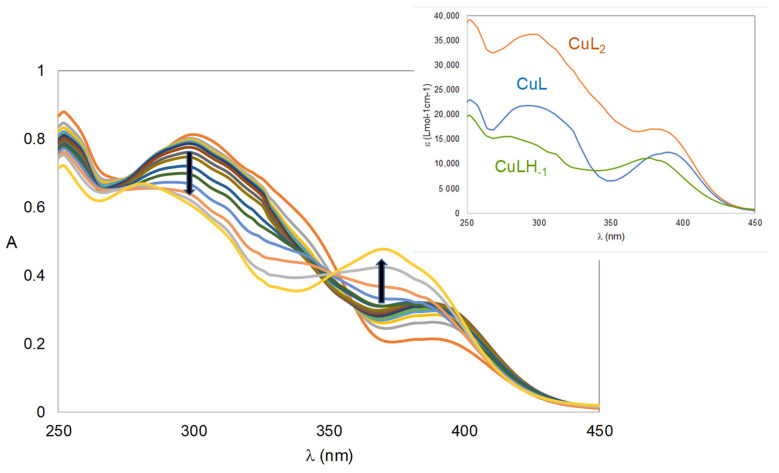
Absorbance spectra for the Cu(II)/**5a** 1:2 system (2.82 < pH < 9.43) in 50% (*w*/*w*) DMSO/H_2_O (*C*_L_ = 4 × 10^−5^ M) and outset with individual calculated spectra by Psequad [31]. The arrows indicate the evolution of the spectra when pH increases. Each line corresponds to an absorbance spectrum obtained at a specific pH.

The calculated spectra contained in Figure 4 for the 1:2 Cu(II)/**5a** system show that CuL and CuL_2_ species have somewhat similar spectra, but the intensity is higher for CuL_2_, which displays λ_max_ (ε, Lmol^−1^ cm^−1^) values corresponding to 298 nm (3.6 × 10^4^) and 384 nm (1.7 × 10^4^). For CuH_−1_L, a complex with an additional coordinated site occupied with a hydroxyl group, a spectrum less intense than that for CuL is observed, which can point towards the possibility of the existence of some slight precipitation. Concerning the (1:3) Fe(III)/**5a** system, Figure S1 confirms, as expected, that there is an intensity increase and blue shift (from 570 to 545 nm) of the bands of the calculated spectra when going from FeL to FeL_3_.

**Figure 5:**
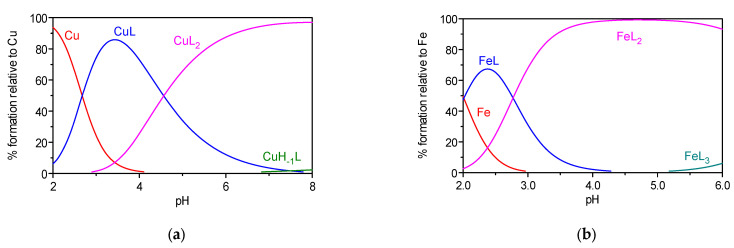
Species distribution curves for the systems: Cu(II)/**5a** 1:2 (**a**) and Fe(III)/**5a** 1:3 (**b**); *C*_L_ = 4 × 10^−5^ M).

Figure 5 shows, as an example, the species distribution diagrams for the (1:2) Cu(II)/**5a** and (1:3) Fe(III)/**5a** systems. For both systems, the species distribution curves reveal that, under the conditions of the spectrophotometric titrations performed, the main species formed above pH 5.5 is ML_2_.

Comparison of the metal-chelating capacities of compounds **4c** and **5a** shows that pFe values (at pH 7.4, *C*_L_/*C*_M_ = 10, *C*_M_ = 10^−6^ M) are similar for both compounds (pFe 16.8–17.4) while for pCu the value obtained for **5a** (13.6) is higher than that obtained for **4c** (10.3). This behavior points toward the possibility of **5a** having stronger copper coordination, likely due to a tridentate coordination mode, resulting from the further involvement of the nearby carbonyl oxygen atom from the linker in the coordination core (see Section 2.1.2). This ability for tridentate (*N*,*O*,*O*) coordination was also previously detected for the copper complexation of analogous positional isomers of DNP–BIM hybrids (pCu 13.9 vs. 10.7 [32]).

#### 2.1.2. Molecular Modeling of the Copper Complexes

Since no X-ray structures were obtained, molecular modeling was performed to obtain further information about the structures of the 1:1 and 1:2 (Cu/L) complexes with the positional hybrids **4a** and **5a**. Compound **4c** was substituted by **4a**, with the expected analogous metal coordination core, in order to keep the same total number of atoms in the complexes and make the molecular modeling comparison easier. 

Concerning the 1:1 (Cu/L) complexes, a bidentate (*N*,*O*) coordination with two coordinating water molecules was considered for the complex with **4a**, while a tridentate (*N*,*O*,*O*) coordination mode with one coordinated water molecule was attributed to the copper complex with **5a**.

**Figure 6:**
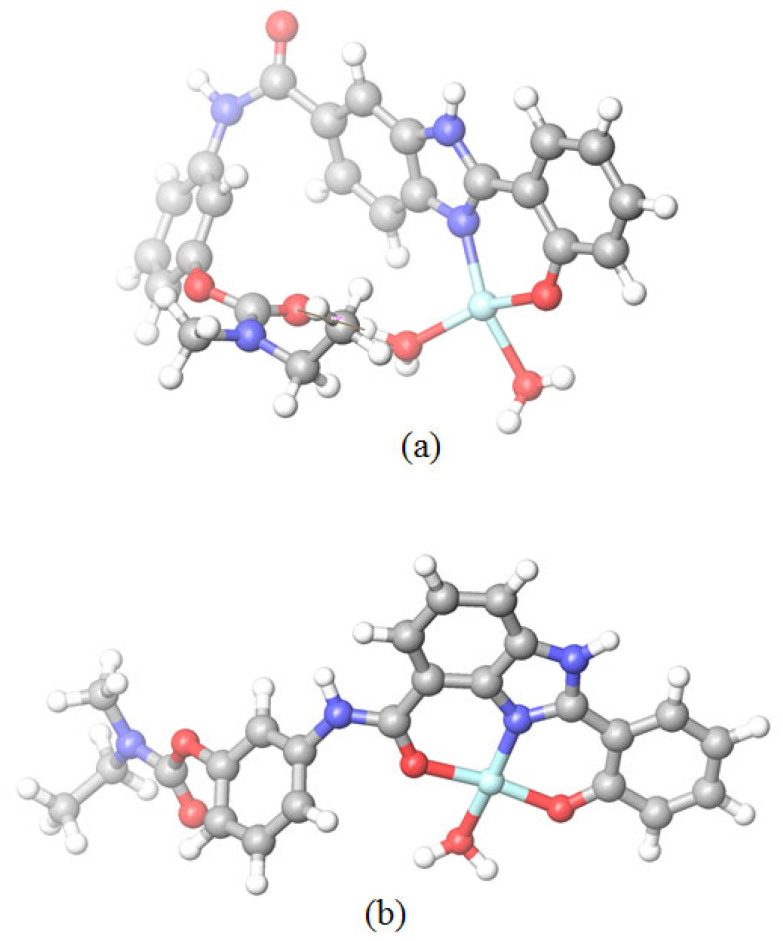
DFT-minimized structures of 1:1 Cu/L complexes with **4a** (**a**) and **5a** (**b**). Color of atoms: Cu (light blue), N (blue), O (red), C (grey), and H (white).

All molecular modeling studies were performed with full geometry optimization of the copper complexes by quantum mechanical calculations grounded on DFT methods included in the Gaussian 03 software package [34], with the B3PW91 function [35]. Figure 6 shows that the energy-minimized structure of the 1:1 copper complex with **4a** corresponds to a distorted tetrahedral while that with **5a** has a distorted square planar geometry.

Although similar copper coordination bond distances can be found for both energy-minimized structures contained in Figure 6, such as Cu-H_2_O (2.005−2.065 Å) and Cu-O-phenol (1.865−1.869 Å), the Cu-N distance decreases from 1.918 to 1.885 Å when going from the complex with **4a** to that with **5a**. This is mostly due to the existence of a tridentate coordination mode in the latter case, which involves the adjacent O-carbonyl (distance Cu-O-carbonyl 1.964 Å). The copper coordination to compound **4a** exhibits a strong distortion relative to a regular tetrahedral geometry (coordination bond angles 109.5°) since copper coordination bond angles are in the range of 82.0−103°. Regarding the copper complex with **5a** (see Figure 6b), the distortion in relation to a regular square planar geometry (coordination bond angles 90°) is detected through its coordination bond angles (87.0−94.1°) and the nearly planar geometry evidenced by the bond angles of O-phenol-Cu-O carbonyl (172°) and N-Cu-water (172.2°).

A comparison of the stability of the copper complexes 1:1 with **4a** and **5a** was made in terms of the determination of the free energy difference (ΔG = −56.4 kcal/mol) between the Cu-**5a** complex plus one water molecule and the Cu-**4a** complex. The obtained results prove that the copper complex with **5a** is more stable than the corresponding one with **4a**, which is in accordance with the previous complexation studies performed in a solution for **4c** (analogous of **4a**) and **5a** (see Section 2.1.1. Metal Chelation). 

**Figure 7:**
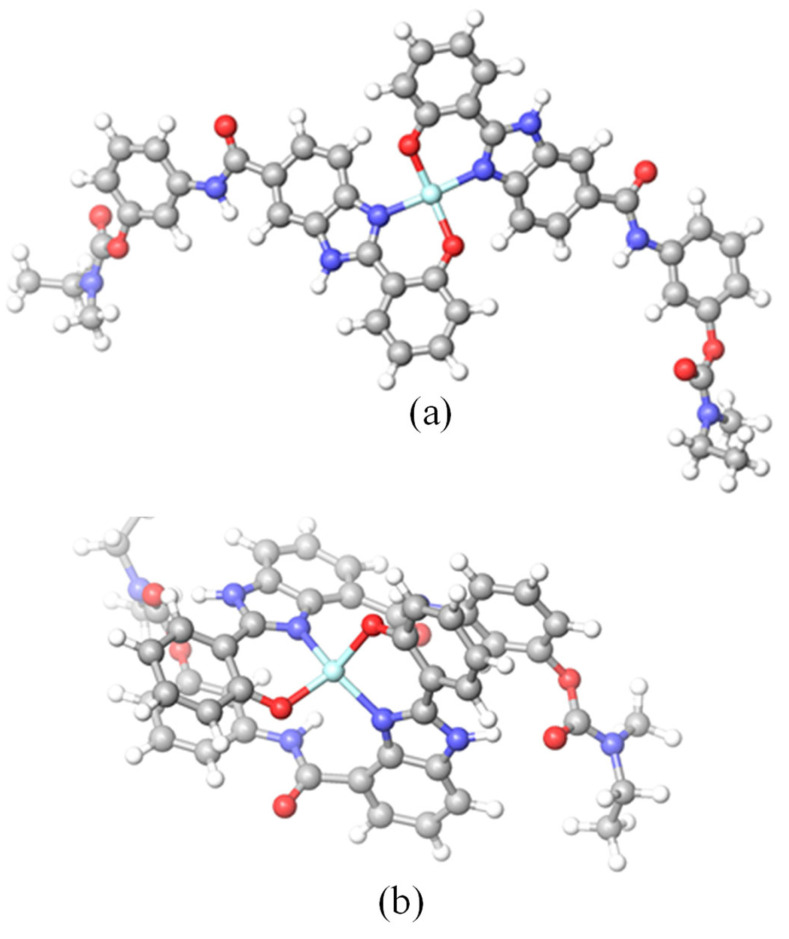
DFT-minimized structures of Cu/L (1:2) complexes with (**a**) **4a** and (**b**) **5a**. Color of atoms: Cu (light blue), N (blue), O (red), C (grey), and H (white).

Regarding the 1:2 Cu/L complexes, Figure 7 provides evidence for both complexes of a distorted four-coordinate geometry, being square planar for the complex with **4a** and intermediate between square planar and tetrahedral for the complex with **5a**. In fact, the great distortion of the square planar coordination geometry of the 1:2 copper complex with **4a** is evident from the values of the coordination bond angles (91.2–92.8°) and the lack of planarity found for the bond angles O-Cu-O (159.4°) and N-Cu-N 158.3°). For the 1:2 copper complex with **5a**, both the coordination bond angles (94.2−100.8°) and the bond angles O-Cu-O (139.8°) and N-Cu-N 139.4°) reveal a higher distortion of the tetra-coordinate geometry, which can be seen as intermediate between square planar and tetrahedral. Nevertheless, similar coordination bond distances were measured for both complexes: Cu-O (1.895−1.923 Å) and Cu-N (1.989−2.005 Å). 

Moreover, the CuL_2_ complex with the twisted compound **5a** has a more compact structure than the linear compound **4a**, which appears more distended. In fact, this can be explained due to the formation of two intramolecular H-bonds between the N-H of the BIM moiety and the carbonyl oxygen atom of the RIV portion of the other ligand (1.911−1.927 Å) in the case of the 1:2 copper complex with **5a**.

Since these two CuL_2_ complexes have an equal number of atoms, the comparison of their relative stability can be performed in terms of the calculated minimum energies. The 1:2 (Cu/L) complex with **4a** was found to be more stable (4.56 kcal/mol) than the one with **5a**, likely due to the less strained copper coordination obtained in the complex with **4a**. 

### 2.2. Biological Studies

#### 2.2.1. Inhibition of A*β* Aggregation and Cu(II) Role

Since the presence of Cu^2+^ can promote A*β* aggregation and Cu-associated events in AD [26], and these RIV–BIM hybrids enclose metal-chelating moieties, it appears interesting to re-analyze how these chelators studied here can interfere in the capacity for A*β* aggregation.

Table 2 contains a summary of the inhibitory results previously obtained for the RIV–BIM hybrids regarding the self- and Cu-induced aggregation of A*β*_42_ by molecular fluorescence spectroscopy [21]. Moreover, all the compounds were less potent inhibitors than the reference polyphenolic compound (curcumin). Interestingly, these compounds are only slightly more effective as inhibitors of copper-induced A*β*_42_ aggregation, as compared to self-mediated aggregation, thus suggesting that the copper-chelating ability does not seem to be the major factor in the aggregation process. In fact, the conditional dissociation constants (*K*’_d_) for the copper complexes calculated at pH 7.4 are 32 nanomolar (**4c**) and 98 picomolar (**5a**), with both values being outside the range of *K*’_d_ = 1–10 picomolar corresponding to chelators eventually able to recover copper from the A*β* peptide (*K*’_d_ = 10 picomolar—100 nanomolar for Cu(A*β*) complexes) [36]. Thus, competition for copper between these hybrids and the A*β* peptide may not be significant, and so the inhibition process must be mostly regulated by the capacity of ligand intercalation between β-sheets of A*β* fibrils.

In summary, it can be concluded that RIV–BIM hybrids of series **5** (**5a**, **5b,** and **5d**) better prevent the Aβ_42_ aggregation than the respective positional isomers of series **4** (**4a**, **4b**, and **4d**). The inhibition process may be primarily dependent on the relative abilities of the compounds to intercalate between fibrils rather than on their copper-chelating capacity.

#### 2.2.2. Inhibition of Monoamine Oxidases

MAO inhibition is a well-recognized target in the design of drugs for AD and PD treatment. MAOs are bound to the outer mitochondrial membrane and play a central role in the metabolism of neurotransmitters such as dopamine, serotonin, and adrenaline. Although MAO-A and MAO-B have 70% sequence identity, they display different substrate and inhibitor characteristics. MAO-A preferentially metabolizes serotonin, while dopamine is metabolized by both isoforms [39]. Some of the currently available MAO inhibitors have an irreversible mechanism of action, such as the aminopropargyl-containing drugs *R*-deprenyl (selegiline) or clorgyline, which are selective for MAO-B or MAO-A, respectively, while in other drugs, such as safinamide, that group is absent, affording selective-reversible mechanisms of action [40].

Selective inhibition of MAO-A in the human brain is associated with the treatment of mental disorders (e.g., anti-depressant), while selective MAO-B inhibitors are those used for treating PD. The efficiency of MAO-A inhibitors is considerable for treating atypical depression, anxiety, bipolar depression, and long-term depression [41,42], attributable to an increase in monoamine-based neurotransmitters at nerve terminals [43], while the MAO-B inhibitors are not effective as anti-depressants since they have no direct effect on serotonin or norepinephrine metabolism. However, ca 70% of PD patients are known to evolve in later stages to dementia (PDD) [44], which generally presents similar features to AD dementia, and so some common pharmacological symptomatic treatments have been used. In particular, rivastigmine, the dual cholinesterase (AChE-BuChE) inhibitor anti-AD drug, has also been approved by the US FDA for the treatment of mild-to-moderate PD dementia [45,46].

Table 2 summarizes the results of human (*h*) MAO inhibition screenings with RIV–BIM hybrids **4a**–**d** and **5a**–**b** and the reference MAO-A and MAO-B inhibitors (clorgyline and selegiline, respectively). 

The RIV–BIM hybrids, such as **4a**, **4b**, and **4d**, presented *h*MAO inhibitory activity selective for isoform A, showing IC_50_ values within the low µM/high nM range. The position of the carboxamide group at the BIM moiety is important for MAO-A inhibition. In fact, while compounds containing the carboxamide group at position **5** of the heterocycle (compounds **4a** and **4b**) were able to inhibit MAO-A, the same did not apply to the 4-substituted analogues (compounds **5a** and **5b**, respectively). Moreover, the *h*MAO-A inhibitory activity decreased with the increase in the number of carbons between the carboxamide group and the rivastigmine-based fragment (IC_50_ compound **4a** < compound **4b** < compound **4c**). 

With the aim of aiding the rationalization of these experimental results, docking modeling studies were performed, involving a selection of hybrids (**4a**, **4d**, and **5a**). 

#### 2.2.3. Docking Studies of Selected Hybrids with *h*MAOs 

**Figure 8:**
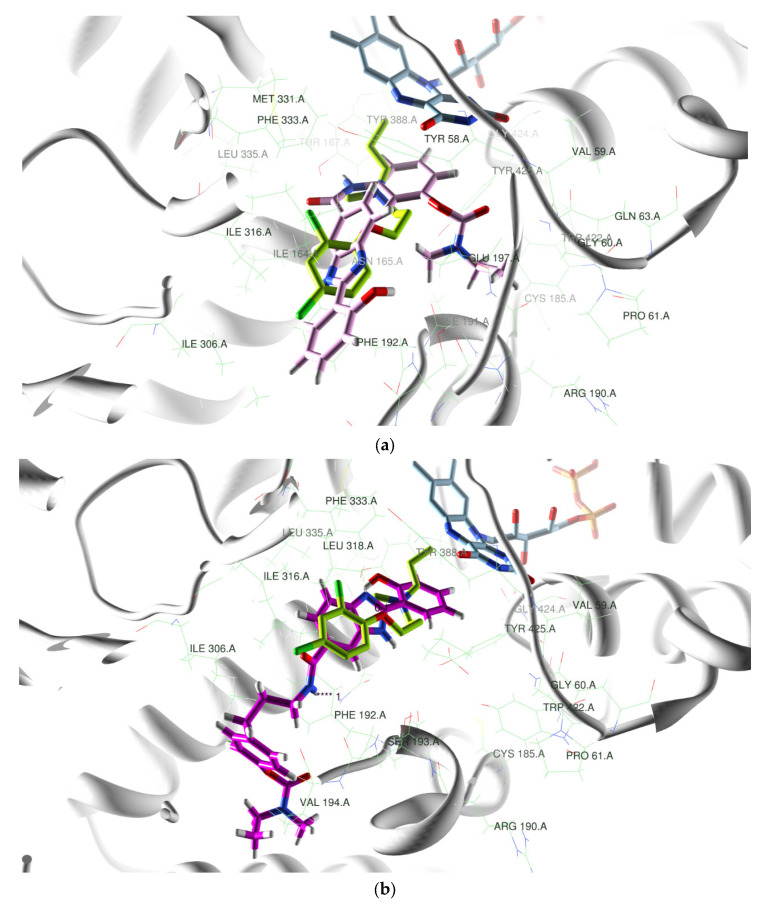

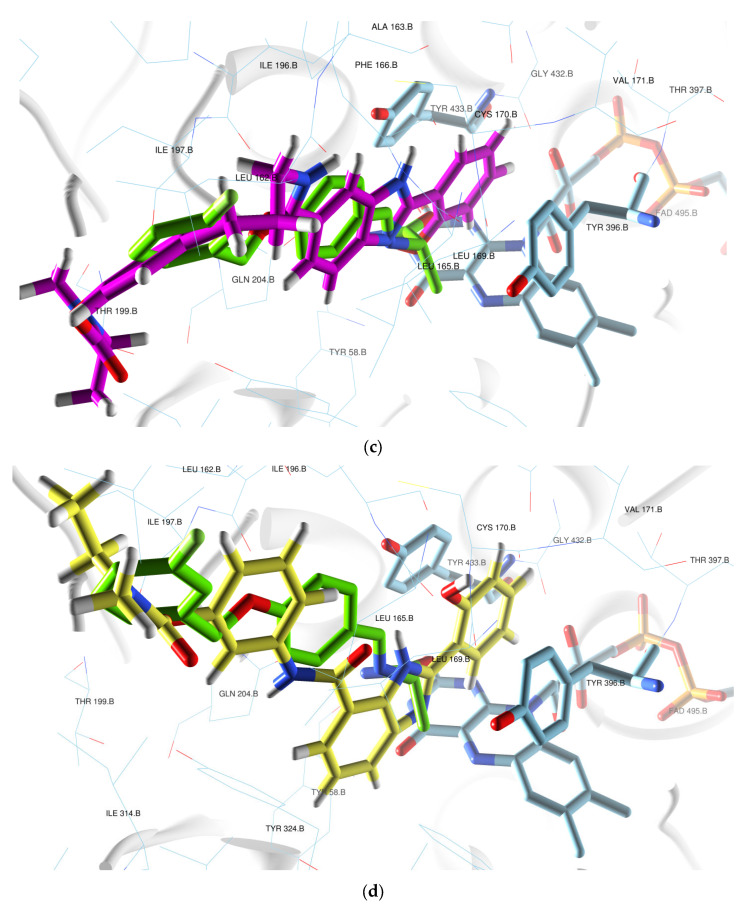
Superimposition of original ligand (clorgyline, green carbon chain) (PDB code: 2BXR) with (**a**) **4a** (pink) and (**b**) **4d** (magenta), inside the *h*MAO-A active site. Superimposition of original ligand (safinamide, green carbon chain) (PDB code: 2V5Z) with (**c**) **4d** (magenta) and (**d**) **5a** (yellow), inside the *h*MAOB active site. FAD and residues near this cofactor with light blue carbon chains.

In order to corroborate the biological activity of the RIV–BIM hybrids as inhibitors of the FAD (flavine-adenine-dinucleotide)-dependent enzymes MAO-A and MAO-B, a representative selection of compounds was investigated by computational molecular docking to the active site of these enzymes. The representative selection (**4a**, **4d**, and **5a**) was chosen to obtain some insight into the effect of different BIM positional isomers (**4a** vs. **5a**) and different sizes of the linker between the RIV and the BIM moieties (**4a** vs. **4d**). As the first step of this simulation, the models of the original ligands (clorgyline or safinamide) of the X-ray structure of the corresponding complex with *h*MAO-A or *h*MAO-B, respectively, were re-docked into the active site cavity of the enzyme (PDB code: 2BXR [47] and 2V5Z [48]). Subsequently, the docking of the hybrids was carried out, and visualization of their best docking poses inside the active site cavity of *h*MAO-A and *h*MAO-B is shown in Figure 8 and Figure S2.

The *h*MAO-A’s active site is composed of a unique hydrophobic cavity (ca 550 Å^3^), while *h*MAO-B has a bipartite cavity, enclosing the active site cavity (~400 Å^3^) and a smaller entrance cavity (~300 Å^3^) gated by both Ile and Tyr residues [48]. Therefore, the MAO-A isoform is expected to better accommodate bulkier ligands than the MAO-B isoform due to its small entrance cavity.

For the docking with *h*MAO-A, since the X-ray structure of its complex with clorgyline involves a covalent adduct (with the linkage of the flavin N5 atom to the amine-propargyl group), that link was disrupted, and then missing atoms, side chains, and FAD bonding orders were fixed in the original PDB structure to obtain the model of the *h-*MAO-A structure. Figure 8 and Figure S2 show that the three RIV–BIM ligands adopted quite different conformational structures and accommodations inside the active site of *h-*MAO-A. In particular, they faced the FAD in a different manner as follows: **4a** with the phenyl group of the RIV moiety (distance C-H to FAD (O4) 3.9 Å); **4d** with the hydroxyphenyl group of the BIM portion (distance O-H to FAD (O4) 3.9 Å); and **5a** with the phenyl group of the benzimidazole BIM moiety) (distance C-H to FAD (O4) 1.4 Å), while the corresponding hydroxyphenyl group is pointed away from FAD. Inside the active site, both compounds **4a** and **5a** are bent relative to the original ligand, with a close parallel positioning of the RIV and BIM moieties, respectively, relative to the FAD. In opposition, compound **4d** is aligned with the original ligand. Moreover, compound **4a** establishes two H-bonds, between the NH acetamide group amid the RIV and BIM moieties and TYR407, as well as between the carboxylic oxygen of the RIV moiety and GLN74; compound **5a** establishes only one H-bond between the hydroxyl group of the BIM moiety and GLN74. 

Therefore, concerning *h*MAO-A inhibition, although compound **5a** is nearer to the FAD than the other two compounds, these two compounds seem to establish more convenient interactions inside the active site of the enzyme through H-bonds (**4a**) or better superimposition with the original ligand (**4d**), in accordance with the results obtained in Table 2 for *h*MAO-A inhibitory activity.

Regarding *h*MAO-B inhibition, the ligand–enzyme binding interactions found herein seem to be primarily governed by the variation of the steric, electrostatic, hydrophobic, and H-bond interactions with the FAD cofactor and other relevant amino acid residues inside the enzyme active site, similar to what was reported for safinamide (a selective and reversible inhibitor drug approved by the European Medicines Agency (EMA) in 2017 for the treatment of PD [49]) and other MAO inhibitors without an aminopropargyl group [40], but, unlike most of the amine-propargylated MAO inhibitors, they are not covalently bound to FAD. In fact, Figure 8 and Figure S2 show that these compounds (**4a**, **4d**, and **5a**), similar to the reference ligand safinamide, do not form a covalent adduct with the flavin cofactor inside the *h*MAO-B active site and present an extended conformation that occupies the two cavities of the enzyme: The substrate cavity, in front of the flavin, and the entrance cavity below the protein surface. Inside the substrate cavity of the *h*MAOB active site, all the compounds face the FAD with the BIM moiety, with the hydroxybenzyl group establishing π-stacking with both TYR433 and TYR396. The hydroxyl group of the BIM moiety points to the FAD for compounds **4a** and **4d**, with the distance between it and the carbonyl in FAD (495 O4) being 2.7 Å and 3.1 Å, respectively. In the case of compound **5a**, the hydroxyl group points in the opposite direction, and the distance between the nearest C-H group of the hydroxybenzyl and the carbonyl in FAD (495 O4) is 3.5 Å.

H-bonds are also established near the BIM moiety for compounds **4a** (one among the hydrogen of the NH carboxamide group between the RIV and BIM moieties and TYR324) and **5a** (two between the hydrogen of the hydroxyl group of BIM and CYS170), while for **4d**, the carboxylic oxygen of the RIV moiety is engaged in an H-bond with TYR324, in the entrance cavity.

Taking into consideration the *h*MAO-B inhibitory results shown in Table 2, it can be concluded that the position of the hydroxyl group relative to the FAD is not an absolute determinant for the enzyme inhibition capacity, but the establishment of H-bonds with the hydroxyl group of BIM or with the carboxylic oxygen of the RIV moiety seems to contribute positively to its inhibition potential.

#### 2.2.4. Cell Viability and In Vitro Neuroprotection 

In the early 1980s, it was discovered that 1-methyl-4-phenyl-1,2,3,6-tetrahydropyridine (MPTP) caused a syndrome in humans that resembles Parkinson’s disease (PD) [50]. MPTP was shown to cross the blood–brain barrier and to be metabolized to 1-methyl-4-phenyl-pyridinium (MPP^+^) in glial cells of the brain. It is then taken up by dopaminergic neurons via the dopamine transporter (DAT), where it accumulates in the mitochondria and inhibits complex I of the electron transport chain, causing cell death and oxidative damage [50].

**Figure 9:**
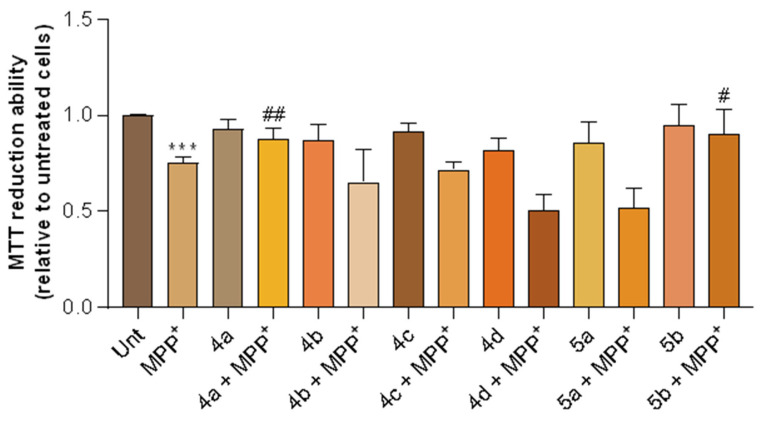
Neuroprotective effects of RIV–BIM compounds against MPP^+^-induced toxicity in SH-SY5Y cells. Cells were treated with the compounds for 1 h and then MPP^+^ was added to the medium for 24 h. MTT reduction assay was performed to assess cell viability, and the results are expressed relative to those for SH-SY5Y untreated cells. *** *p* < 0.001, significantly different when compared with SH-SY5Y untreated cells; ## *p* < 0.01, significantly different when compared with MPP^+^-treated SH-SY5Y cells; # *p* < 0.05, significantly different when compared with MPP^+^-treated SH-SY5Y cells. Statistical differences were analyzed using one-way ANOVA followed by Tukey’s post hoc test or using Student’s *t*-test.

The neuroblastoma SH-SY5Y cell line has been widely used as an in vitro model of dopaminergic neurons in PD research [51], because of its similarities with dopaminergic neurons; these cells possess the ability to synthesize dopamine (DA) and norepinephrine and they express DAT, a protein that regulates DA homeostasis and is responsible for MPP^+^ incorporation into neurons [52]. Therefore, when treated with MPP^+^, SH-SY5Y cells mimic several aspects of the dopaminergic neuron death observed in PD [52]. For the aforementioned reasons listed above, and in order to study the neuroprotective effects of RIV–BIM compounds, SH-SY5Y cells were treated with MPP^+^, a neurotoxic metabolite of the Parkinsonian-inducing agent 1-methyl-4-phenyl-1,2,3,6-tetrahydropyridine (MPTP) [53]. Indeed, this neurotoxin induced a decrease of approximately 30% in cell viability when compared with untreated cells (Figure 9). Interestingly, our results revealed significant protection of two compounds, namely **4a** and **5b**, on MPP^+^-induced toxicity in SH-SY5Y cells (Figure 9). These compounds prevented cell toxicity induced by MPP^+^, thus suggesting that they may have a potential therapeutic effect on PD.

## 3. Materials and Methods

### 3.1. Metal Complexation Studies

#### 3.1.1. Materials and Equipment

Analytical-grade reagents were acquired from suppliers and used without additional purification. The aqueous iron(III) (0.0177 M) and copper(II) (0.015 M) stock solutions were prepared from 1000 ppm Titrisol standards and their rigorous metal concentration was assessed by flame atomic absorption spectroscopy. The 0.1 M HCl solution used for the calibration of the glass electrode was prepared from a Titrisol ampoule. The iron stock solution was prepared in acid excess, to avoid hydrolysis, and its acid content was determined by the standard-addition method using 0.1 M HCl (Titrisol). The titrant (KOH solution) used was prepared from carbonate-free commercial concentrate solutions (Titrisol, KOH 0.1 M ampoules). The KOH solution was standardized by titration with potassium hydrogen phthalate solution and discarded when the percentage of carbonate, determined by Gran’s method [53], was greater than 0.5% of the total amount of base. The spectrophotometric titrations were carried out by using a Crison micropH 2002 milivoltimeter, a Crison microBu 2031 burette, a Haake thermostatic bath (*T* = 25.0 ± 0.1 °C), and a Perkin-Elmer (Waltham, MA, USA) Lambda 35 spectrophotometer.

#### 3.1.2. Spectrophotometric Titrations

The two positional isomeric hybrids **4c** and **5a** were titrated, alone or in the presence of the metal ions, in a 50% *w*/*w* DMSO/H_2_O medium, at *T* = 25.0 ± 0.1 °C and ionic strength of (I) 0.1 M KCl, by using a 0.1 M KOH (50% *w*/*w* DMSO/H_2_O) titrant solution. The glass and Ag/AgCl reference electrodes were previously conditioned in different DMSO/H_2_O mixtures of increasing DMSO % compositions, and the response of the glass electrode was evaluated by strong acid–strong base (HCl/KOH) calibrations with the determination of the Nernst parameters by Gran’s method [54]. The measurements were performed in a final volume of 30.00 mL and the ligand concentration (C_L_) was 4 × 10^−5^ M, under different C_M_/C_L_ (M = Fe, Cu) proportions: 0:1 (L), 1:1, 1:2, and 1:3. The spectrophotometric measurements were carried out in a 250–450 nm (L, Cu/L systems) or 250–650 nm (L, Fe/L systems) wavelength range at pH ca 2.5–8.0. Under these specified experimental conditions, the p*K*w value (14.95) was determined and subsequently used in the calculations. The stepwise protonation constants of the ligands, *K*_i_ = [H_i_L]/[H_i−1_L][H], and the overall metal–complex stability constants, $\beta_{M_{m}H_{h}L_{l}}$ = [M_m_H_h_L_l_]/[M]^m^[H]^h^[L]^l^, were calculated by fitting the spectrophotometric data with the Psequad [31] program. The metal hydrolysis model was determined under the established experimental conditions (*I* = 0.1 M KCl, 50% *w*/*w* DMSO/H_2_O, *T* = 25.0 ± 0.1 °C) and the following values of stability constants were included in the equilibrium models related to the Fe(III)/L and Cu(II)/L systems: log $\beta_{{\mathrm{FeH}}_{-1}}$ = −4.07, log $\beta_{{\mathrm{FeH}}_{-3}}$ = −11.42; log $\beta_{{\mathrm{Cu}}_{2}H_{-2}}$ = −9.99. The species distribution curves were obtained with the Hyss program [55].

#### 3.1.3. ^1^H NMR Studies

The proton NMR titration of compound **4c** (*C*_L_ = 4.5 mM) in a 75% *d*_6_-DMSO/D_2_O medium was carried out by using DCl or CO_2_-free KOD solutions and an Orion Star Thermo Scientific instrument fitted with a combined Mettler Toledo U402-M3-S7/200 microelectrode to ascertain pH* values (reading of the pH meter previously calibrated with standard aqueous buffers pH 4 and 7) [56]. This ^1^H NMR titration was only used to confirm the sequence of protonation of the compounds. This study involved the use of a slightly different working medium (75% *d*_6_-DMSO/D_2_O instead of 50% DMSO/H_2_O), due to the required higher concentration of ligand for NMR measurements, but it does not have further implications in the projected outcome of this study.

#### 3.1.4. Molecular Modeling of the Cu(II) Complexes

Since X-ray structures of the copper complexes were not available, a molecular modeling study was performed to obtain some prevision of the coordination core for the 1:1 and 1:2 (Cu/L) complexes with hybrids **4a** and **5a**. One and two water molecules were included in the coordination shell of the 1:1 copper complexes with compounds **5a** and **4a**, respectively. These studies were performed with full geometry optimization of the complexes by quantum mechanical calculations based on DFT methods, included in the Gaussian 03 software package [34], with the B3PW91 hybrid functional [35]. The initial molecular simulation was accomplished by using the 3-21G basis set [57] and afterwards 6-31G** [58], with no symmetry constraints being imposed during geometry optimization with both basis sets. Analogous calculations were also achieved for one water molecule. Concerning the 1:1 copper complexes, the electronic energies (*E*b1) obtained at the PBE0/b1 level of theory were converted to free energy (*G*b1) at 298.15 K and 1 atm by using zero-point energy and thermal energy corrections based on structural and vibration frequency data calculated at the same level.

### 3.2. Biological Assays

#### 3.2.1. Inhibition of Monoamine Oxidase

The inhibitory activity of the rivastigmine hybrids (RIV–BIM) on *h*MAO-A and *h*MAO-B was studied using an experimental protocol described elsewhere [59] with some modifications [38]. The outlined *h*MAO inhibition was assessed in microsomal MAO isoforms prepared from insect cells (BTI-TN-5B1-4) infected with recombinant baculovirus containing cDNA inserts for *h*MAO-A or *h*MAO-B (Sigma-Aldrich Quimica S.A., Madrid, Spain) and by measuring the enzymatic conversion rates of kynuramine into 4-hydroxyquinoline. The appropriate amounts of *h*MAO-A and *h*MAO-B were adjusted to obtain, in our experimental conditions, the same maximum velocity (*V*_max_ = 50 pmol.min^−1^) for both isoforms (*h*MAO-A: 3 ng mL^−1^; *h*MAO-B: 12 ng mL^−1^). All assays were performed under sodium phosphate-buffered conditions (50 mM, pH = 7.4). The compounds under study and reference inhibitors were pre-incubated at 37 °C for 10 min in the presence of kynuramine (*K*_m_ (*h*MAO-A) = 20 mM; *K*_m_ (*h*MAO-B) = 20 mM; final concentration: 2 × *K*_m_) in 96-well microplates (BRAND plates, pureGradeTM, BRAND GMBH, Wertheim, Germany). Then, the reaction was started with the addition of *h*MAO-A or *h*MAO-B. Initial velocities were determined spectrophotometrically in a microplate reader (Biotek Synergy HT, Winooski, VT, USA) at 37 °C by measuring the formation of 4-hydroxyquinoline at 316 nm, over a period of at least 30 min (interval of 1 min). Data were analyzed using GraphPad PRISM version 6 for Windows (GraphPad Software^®^, San Diego, CA, USA). The initial velocities, obtained from the linear phase of product formation, were normalized and plotted against the respective inhibitor concentration. IC_50_ values were obtained from dose–response curves and were expressed as the mean ± standard deviation. IC_50_ values were determined from at least three independent experiments, each performed in triplicate.

#### 3.2.2. Cell Viability and In Vitro Neuroprotection

SH-SY5Y human neuroblastoma cells were kept in culture under a humidified atmosphere of 5% CO_2_ at 37 °C in Dulbecco’s Modified Eagle Medium/Nutrient Mixture F-12 (DMEM/F-12) (Gibco, Thermo Fisher Scientific) supplemented with 10% heat-inactivated fetal bovine serum (FBS), 50 U/mL penicillin, and 50 μg/mL streptomycin. Cells were plated at a density of 1 × 10^5^ cells/mL and pre-incubated for 1 h with the compounds by adding them to the medium at the following final concentrations: 1 µM for compounds **4a**, **4b,** and **5b**; 2 µM for compounds **4c** and **5a**; and 3 µM for compound **4d**. Then the cells were incubated for 24 h with MPP^+^ (1-methyl-4-phenylpyridinium, Sigma-Aldrich) (1 h pre-incubation + 24 h incubation) at the final concentration of 0.5 mM. MPP^+^ was freshly prepared in sterile water on the day of the experiment. The compounds were dissolved in DMSO, aliquoted, and stored at −20 °C; their toxicity was previously studied through cell viability assays to select the highest non-toxic concentration of each compound.

Cell viability was measured by the colorimetric MTT (3-(4,5-dimethylthiazol-2-yl)-2,5-diphenyltetrazolium bromide) reduction assay, which follows the principle that viable cells can metabolize MTT into formazan that absorbs light at 570 nm. After the cell treatments with the compounds and MPP^+^, the culture medium was removed, 150 µL of MTT (0.5 mg/mL dissolved in sodium medium) was added to each well, and the 48-well plate was incubated at 37 °C for 2 h. The formazan precipitates were then solubilized with 150 µL of 0.04 M HCl/isopropanol. The absorbance was read at 570 nm using a spectrophotometer (Spectramax Plus 384), and cell viability results were normalized to untreated SH-SY5Y cells, with the means ± SEMs derived from six different experiments performed in duplicate (*N* = 6). Statistical analyses were performed on GraphPad Prism 8 (GraphPad Software, San Diego, CA, USA) using one-way analysis of variance (ANOVA) followed by Tukey’s post hoc test or using Student’s *t*-test to determine statistical significance.

### 3.3. Molecular Docking of Monoamine Oxidases

The model for the *h*MAO isoforms was obtained from the RCSB Protein Data Bank (PDB) X-ray crystallographic structures, namely from enzyme–ligand complexes with PDB codes 2BXR [47] and 2V5Z [48] for *h*MAO-A and *h*MAO-B, respectively. The model structures of the compounds **4a**, **4d**, and **5a** were obtained by Maestro v9.3 [60], and their geometry was firstly optimized using Ghemical v. 2.0 [61] and then submitted to a random conformational search (RCS) of 1000 cycles and 2500 optimization steps using the Tripos 5.2 force field [62]. To obtain the MAO´s structure, the original PDB complex model structures were treated using Maestro, by removing the original ligand, solvent, and co-crystallization molecules and then adding the H atoms. In the case of the structure with PDB code 2BXR, the clorgyline ligand was covalently bonded to FAD, and so missing atoms, side chains, and bonding orders of FAD were fixed.

The minimized structures of the ligands were docked into the *h*MAO´s structure with GOLD software v. 5.2 [63], and the zone of interest was established as the residues within 5 Å from the original position of the ligand in the crystal structure. The ‘allow early termination’ option was deactivated, and the remaining default parameters of Gold were used. The ligands were subjected to 100 genetic algorithm steps using ASP as the fitness function, and the best 5 poses per ligand were retained. The docking protocol was validated by re-docking the co-crystallized original ligands into the corresponding active sites of the *h*MAOs isoforms.

## 4. Conclusions

Alzheimer’s Disease (AD) and Parkinson´s Disease (PD) are complex neurodegenerative illnesses known for their multifactorial origin, with an urgent need for effective disease-modifying drugs. Cholinesterases (Che: AChE, BChE) and the amyloid-*β* peptide (A*β*) are among the most important targets in AD therapy. The newly developed set of multifunctional Rivastigmine–Benzimidazole hybrids (RIV–BIM) demonstrated good capacity for the inhibition of one or both ChE and also the A*β* peptide aggregation. The ChE inhibitory activity is primarily attributed to the RIV moiety, while the anti-Aβ aggregation capacity and the antioxidant activity are primarily ascribed to the BIM moiety and the inherent substituent groups, namely with metal-chelating capacity. Given that the inhibition of A*β* aggregation was dependent, at least partially, on the co-presence of Cu(II), and considering the dyshomeostasis of redox-active metal ions (e.g., Cu(II) and Fe(III)) found in some regions of AD and PD patient brains, the metal-chelating capacity for these biometal ions was evaluated in solution and corroborated by computational studies. Notably, the effect of the positional isomeric structure on the Cu(II)-chelating capacity is higher for series **5** than for series **4**, attributed to the corresponding higher denticity of these compounds. Furthermore, since the loss of monoamine neurotransmitters (e.g., Dopamine) is of pivotal importance in PD and AD patient brains, attributed to monoamine oxidases (MAOs: MAO-A and MAO-B), therapeutic agents also involve MAO inhibitors. Therefore, this set of hybrids was assessed in vitro and in silico (docking simulations) for their inhibitory capacity of these enzymes and corresponding selectivity, showing reasonably good results, at least comparable to some single-target drug candidates under phase III clinical trials. Finally, similarly to former studies in neuroblastoma cell lines (SH-SY5Y), which showed that some hybrids (e.g., **4d** and **5a**) could reduce the toxicity induced by A*β* and ROS, as an AD model, identical neuroprotective capacity was herein observed for some compounds (e.g., **4a**, **5b**), when cytotoxicity was induced by MPP^+^, a PD model.

Overall, these RIV–BIM hybrids revealed the capacity to tackle multiple pathological targets of AD and PD, thus deserving further investigation as potential drug candidates for these neurodegenerative diseases.

## Figures and Tables

**Table 1:** Stepwise protonation constants of compounds **4c** and **5a**, global formation constants ^a^ for their Cu(II) and Fe(III) complexes, and pM ^b^ values (*T* = 25.0 ± 0.1 °C, *I* = 0.1 M KCl, 50% *w*/*w* DMSO/water).

Compound	M_m_H_h_L_l_ (mhl)	log *K*_i_	$\log\beta_{Cu_{m}H_{h}L_{l}}$	$\log\;\beta_{Fe_{m}H_{h}L_{l}}$
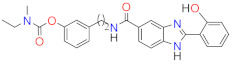	(011)	8.98(1)		
	(021)	3.48(2)		
	(101)		9.09(5)	11.57(6)
	(1–11)		3.40(5)	-
	(102)		17.16(8)	20.35(7)
	(103)		-	28.40(6)
**4c**		**pM**	**10.3**	**16.8**
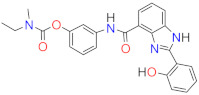	(011)	8.67(1)		
	(021)	3.38(6)		
	(101)		11.30(2)	12.53(5)
	(1–11)		3.82(8)	-
	(102)		20.43(5)	23.82(4)
	(103)		-	30.20(8)
**5a**		**pM**	**13.6**	**17.4**

^a^$\beta_{M_{m}H_{h}L_{l}}$ = [M_m_H_h_L_l_]/[M]^m^[H]^h^[L]^l^; ^b^ pM = −log[M] at pH 7.4 (*C*_L_/*C*_M_ = 10, *C*_M_ = 10^−6^ M) [33].

**Table 2:** Inhibition capacity of A*β*_42_ aggregation and MAOs inhibitory activity of the RIV–BIM hybrids (**4a**–**d** and **5a**–**d**) and reference compounds.

Compounds	A*β*_42_ Self- Aggreg. Inhib. ^a^ (%)	A*β*_42_ Cu-ind. Aggreg. Inhib. ^a^ (%)	IC_50_ (µM) or % Inhibition (at 10 µM)	
			*h*MAO-A	*h*MAO-B
**4a**	39.0	41.2	0.74 ± 0.07	28%
**4b**	39.6	48.8	8.6 ± 0.9	43%
**4c**	21.2	22.0	30%	33%
**4d**	20.1	21.3	11 ± 1	43%
**5a**	44.5	45.4	33%	31%
**5b**	58.7	60.8	22%	34%
**5d**	42.1	40.3	–	–
Curcumin	65.7	62.7	–	–
Safinamide	–	–	13%	25.3 ^b^
R-(–)-Deprenyl ^c^ (Selegiline)	–	–	20 ± 2	0.039 ± 0.004
Clorgyline ^c^	–	–	0.0027 ± 0.0005	2.2 ± 0.3

^a^ Percent of inhibition of Aβ_42_ aggregation in the presence or absence of copper (40 M) and 20 M inhibitor (thioflavin-T fluorescence method). The values are the mean of two independent measurements in duplicate (SEM < 10%) [21]; ^b^ IC_50_(nM) [37]; ^c^ IC_50_ [38].

## Data Availability

Data are contained within the article and Supplementary Files.

## Supplementary Materials

The following supporting information can be downloaded at: https://www.mdpi.com/article/10.3390/ijms24098312/s1.

## References

[1] Alzheimer’s Association (2021). Alzheimer’s disease facts and figures. Alzheimer’s Dement..

[2] Dorsey E.R., Elbaz A., Nichols E., Abbasi N., Abd-Allah F., Abdelalim A., Adsuar J.C., Ansha M.G., Brayne C., Choi J.-Y.J. (2018). Global, regional, and national burden of Parkinson’s disease, 1990–2016: A systematic analysis for the Global Burden of Disease Study 2016. Lancet Neurol..

[3] Tricco A.C., Ashoor H.M., Soobiah C., Rios P., Veroniki A.A., Hamid J.S., Ivory J.D., Khan P.A., Yazdi F., Ghassemi M. (2018). Comparative effectiveness and safety of cognitive enhancers for treating Alzheimer’s disease: Systematic review and network metaanalysis. J. Am. Geriatr. Soc..

[4] Hampel H., Mesulam M.M., Cuello A.C., Khachaturian A.S., Vergallo A., Farlow M.R., Snyder P.J., Giacobini E., Khachaturian Z.S. (2019). Revisiting the cholinergic hypothesis in Alzheimer’s disease: Emerging evidence from translational and clinical research. J. Prev. Alzheimers Dis..

[5] LeWitt P.A., Fahn S. (2016). Levodopa therapy for Parkinson disease: A look backward and forward. Neurology.

[6] Bortolato M., Chen K., Shih J.C. (2008). Monoamine oxidase inactivation: From pathophysiology to therapeutics. Adv. Drug Deliv. Rev..

[7] Pisani L., Catto M., Leonetti F., Nicolotti O., Stefanachi A., Campagna F., Carotti A. (2011). Targeting monoamine oxidases with multipotent ligands: An emerging strategy in the search of new drugs against neurodegenerative diseases. Curr. Med. Chem..

[8] Sweeney P., Park H., Baumann M., Dunlop J., Frydman J., Kopito R., McCampbell A., Leblanc G., Venkateswaran A., Nurmi A. (2017). Protein misfolding in neurodegenerative diseases: Implications and strategies. Transl. Neurodegener..

[9] Barnham K.J., Bush A.I. (2014). Biological metals and metal-targeting compounds in major neurodegenerative diseases. Chem. Soc. Rev..

[10] Liu Y., Nguyen M., Robert A., Meunier B. (2019). Metal ions in Alzheimer’s disease: A key role or not?. Acc. Chem. Res..

[11] De Ricco R., Valensin D., Dell’Acqua S., Casella L., Hureau C., Faller P. (2015). Copper(I/II), α/β-Synuclein and amyloid-β: Menage à trois?. ChemBioChem.

[12] Savelieff M.G., Nam G., Kang J., Lee H.J., Lee M., Lim M.H. (2019). Development of multifunctional molecules as potential therapeutic candidates for Alzheimer’s disease, Parkinson’s disease, and amyotrophic lateral sclerosis in the last decade. Chem. Rev..

[13] Santos M.A., Chand K., Chaves S. (2016). Recent progress in repositioning Alzheimer´s disease drugs based on a multitarget strategy. Future Med. Chem..

[14] Ismaili L., Refouvelet B., Benchekroun M., Brogi S., Brindisi M., Gemma S., Campiani G., Filipic S., Agbaba D., Esteban G. (2017). Multitarget compounds bearing tacrine- and donepezil-like structural and functional motifs for the potential treatment of Alzheimer’s disease. Prog. Neurobiol..

[15] Jeřábek J., Uliassi E., Guidotti L., Korábečný J., Soukup O., Sepsova V., Hrabinova M., Kuča K., Bartolini M., Peña-Altamira L.E. (2017). Tacrine-resveratrol fused hybrids as multi-target-directed ligands against Alzheimer’s disease. Eur. J. Med. Chem..

[16] Sampietro A., Perez-Areales F.J., Martinez P., Arce E.M., Galdeano C., Munoz-Torrero D. (2022). Unveiling the multitarget anti-Alzheimer drug discovery landscape: A bibliometric analysis. Pharmaceuticals.

[17] Zhang C., Lv Y., Bai R., Xie Y. (2021). Structural exploration of multifunctional monoamine oxidase B inhibitors as potential drug candidates against Alzheimer’s disease. Bioorg. Chem..

[18] Liu W., Wang Y., Youdim M.B.H. (2022). A novel neuroprotective cholinesterase-monoamine oxidase inhibitor for treatment of dementia and depression in Parkinson’s disease. Ageing Neur. Dis..

[19] Hiremathad A., Keri R.S., Esteves A.R., Cardoso S.M., Chaves S., Santos M.A. (2018). Novel tacrine-hydroxyphenylbenzimidazole hybrids as potential multitarget drug candidates for Alzheimer’s disease. Eur. J. Med. Chem..

[20] Piemontese L., Tomás D., Hiremathad A., Capriati V., Candeias E., Cardoso S.M., Chaves S., Santos M.A. (2018). Donepezil structure-based hybrids as potential multifunctional anti-Alzheimer’s drug candidates. J. Enzym. Inhib. Med. Chem..

[21] Vicente-Zurdo D., Rosales-Conrado N., León-González M.E., Brunetti L., Piemontese L., Pereira-Santos A.R., Cardoso S.M., Madrid Y., Chaves S., Santos M.A. (2022). Novel rivastigmine derivatives as promising multi-target compounds for potential treatment of Alzheimer’s disease. Biomedicines.

[22] Colca J.R., Finck B.N. (2022). Metabolic Mechanisms connecting Alzheimer’s and Parkinson’s diseases: Potential avenues for novel therapeutic approaches. Front. Mol. Biosci..

[23] Ayton S., Lei P., Bush A.I. (2013). Metallostasis in Alzheimer’s disease. Free Radic. Biol. Med..

[24] Chaves S., Várnagy K., Santos M.A. (2021). Recent multi-target approaches on the development of anti-Alzheimer’s agents integrating metal chelation activity. Curr. Med. Chem..

[25] Bautista-Aguilera O.M., Esteban G., Chioua M., Nikolic K., Agbaba D., Moraleda I., Iriepa I., Soriano E., Samadi A., Unzeta M. (2014). Multipotent cholinesterase/monoamine oxidase inhibitors for the treatment of Alzheimer’s disease: Design, synthesis, biochemical evaluation, ADMET, molecular modeling, and QSAR analysis of novel donepezil-pyridyl hybrids. Drug Des. Dev. Ther..

[26] Gerber H., Wu F., Dimitrov M., Osuna G.M.G., Fraering P.C. (2017). Zinc and copper differentially modulate amyloid precursor protein processing by gamma-secretase and amyloid-beta peptide production. J. Biol. Chem..

[27] Li X., Lei P., Tuo Q., Ayton S., Li Q.X., Moon S., Volitakis I., Liu R., Masters C.L., Finkelstein D.I. (2015). Enduring elevations of hippocampal amyloid precursor protein and iron are features of beta-amyloid toxicity and are mediated by tau. Neurotherapeutics.

[28] Kitazawa M., Hsu H.W., Medeiros R. (2016). Copper exposure perturbs brain inflammatory responses and impairs clearance of amyloid-beta. Toxicol. Sci..

[29] Robert A., Liu Y., Nguyen M., Meunier B. (2015). Regulation of copper and iron homeostasis by metal chelators: A possible chemotherapy for Alzheimer’s disease. Acc. Chem. Res..

[30] Chaves S., Hiremathad A., Tomas D., Keri R.S., Piemontese L., Santos M.A. (2018). Exploring the chelating capacity of 2-hydroxyphenyl-benzimidazole based hybrids with multi-target ability as anti-Alzheimer’s agents. New J. Chem..

[31] Zekany L., Nagypal I. (1985). PSEQUAD. Computational Methods for the Determination of Formation Constants.

[32] Costa M., Josselin R., Silva D., Cardoso S.M., May N., Chaves S., Santos M.A. (2020). Donepezil-based hybrids as multifunctional anti-Alzheimer’s disease chelating agents: Effect of positional isomerization. J. Inorg. Biochem..

[33] Raymond K.N., Carrano C.J. (1979). Coordination chemistry and microbial iron transport. Acc. Chem. Res..

[34] Frisch M.J., Trucks G.W., Schlegel H.B., Scuseria G.E., Robb M.A., Cheeseman J.R., Montgomery J.A., Vreven T., Kudin K.N., Burant J.C. (2004). Gaussian 03, Revision C.02.

[35] Perdew J.P., Burke K., Wang Y. (1996). Generalized gradient approximation for the exchange-correlation hole of a many-electron system. Phys. Rev. B.

[36] Faller P., Hureau C. (2009). Bioinorganic chemistry of copper and zinc ions coordinated to amyloid-peptide. Dalton Trans..

[37] Mesiti F., Maruca A., Silva V., Rocca R., Fernandes C., Remião F., Uriarte E., Alcaro S., Gaspar A., Borges F. (2021). 4-Oxoquinolines and monoamine oxidase: When tautomerism matters. Eur. J. Med. Chem..

[38] Chavarria D., Cagide F., Pinto M., Gomes L.R., Low J.N., Borges F. (2019). Development of piperic acid-based monoamine oxidase inhibitors: Synthesis, structural characterization and biological evaluation. J. Mol. Struct..

[39] Youdim M.B.H., Edmondson D., Tipton K.F. (2006). The therapeutic potential of monoamine oxidase inhibitors. Nat. Rev. Neurosci..

[40] Carradori S., Petzer J.P. (2014). Novel monoamine oxidase inhibitors: A patent review (2012–2014). Expert Opin. Ther. Pat..

[41] Meyer J.H., Wilson A.A., Sagrati S., Miler L., Rusjan P., Bloomfield P.M., Clark M., Sacher J., Voineskos A.N., Houle S. (2009). Brain monoamine oxidase A binding in major depressive disorder: Relationship to selective serotonin reuptake inhibitor treatment, recovery, and recurrence. Arch. Gen. Psychiatry.

[42] Sacher J., Houle S., Parkes J., Rusjan P., Sagrati S., Wilson A.A., Meyer J. (2011). Monoamine oxidase A inhibitor occupancy during treatment of major depressive episodes with moclobemide or St. John’s wort: An [^11^C]-harmine PET study. J. Psychiatry Neurosci..

[43] Bymaster F.P., McNamara R.K., Tran P.V. (2003). New approaches to developing antidepressants by enhancing monoaminergic neurotransmission. Expert Opin. Investig. Drugs.

[44] Hely M.A., Reid W.G., Adena M.A., Halliday G.M., Morris J.G. (2008). The Sydney multicenter study of Parkinson’s disease: The inevitability of dementia at 20 years. Mov. Disord..

[45] Cummings J., Lefevre G., Small G., Appel-Dingemanse S. (2007). Pharmacokinetic rationale for the rivastigmine patch. Neurology.

[46] Kandiah N., Pai M.-C., Senanarong V., Looi I., Ampil E., Park K.W., Karanam A.K., Christopher S. (2017). Rivastigmine: The advantages of dual inhibition of acetylcholinesterase and butyrylcholinesterase and its role in subcortical vascular dementia and Parkinson’s disease dementia. Clin. Interv. Aging.

[47] De Colibus L., Li M., Binda C., Lustig A., Edmondson D.E., Mattevi A. (2005). Three-dimensional structure of human monoamine oxidase A (MAO A): Relation to the structures of rat MAO A and human MAO B. Proc. Natl. Acad. Sci. USA.

[48] Binda C., Wang J., Pisani L., Caccia C., Carotti A., Salvati P., Edmondson D.E., Mattevi M. (2007). Structures of human monoamine oxidase B complexes with selective noncovalent inhibitors: Safinamide and coumarin analogs. J. Med. Chem..

[49] Blair H.A., Dhillon S. (2017). Safinamide: A review in Parkinson’s disease. CNS Drugs.

[50] Chen J., Chen Y., Zheng Y., Zhao J., Yu H., Zhu J., Li D. (2021). Protective effects and mechanisms of procyanidins on Parkinson’s disease in vivo and in vitro. Molecules.

[51] Xicoy H., Wieringa B., Martens G.J. (2017). The SH-SY5Y cell line in Parkinson’s disease research: A systematic review. Mol. Neurodegener..

[52] Xie H.R., Hu L.S., Li G.Y. (2010). SH-SY5Y human neuroblastoma cell line: In vitro cell model of dopaminergic neurons in Parkinson’s disease. Chin. Med. J..

[53] Singer T.P., Ramsay R.R., McKeown K., Trevor A., Castagnoli N.E. (1988). Mechanism of the neurotoxicity of 1-methyl-4-phenylpyridinium (MPP^+^), the toxic bioactivation product of 1-methyl-4-phenyl-1,2,3,6-tetrahydropyridine (MPTP). Toxicology.

[54] Rossotti F.J.C., Rossotti H. (1965). Potentiometric titrations using gran plots: A textbook omission. J. Chem. Educ..

[55] Gans P., Sabatini A., Vacca A. (1996). Investigation of equilibria in solution. Determination of equilibrium constants with the HYPERQUAD suite of programs. Talanta.

[56] Krezel A., Bal W. (2004). A formula for correlating p*K*_a_ values determined in D_2_O and H_2_O. J. Inorg. Biochem..

[57] Dobbs K.D., Hehre W.J. (1987). Molecular orbital theory of the properties of inorganic and organometallic compounds 5. Extended basis sets for first-row transition metals. J. Comp. Chem..

[58] Hariharan P.C., Pople J.A. (1973). The influence of polarization functions on molecular orbital hydrogenation energies. Theor. Chim. Acta.

[59] Hagenow S., Stasiak A., Ramsay R.R., Stark H. (2017). Ciproxifan, a histamine H_3_ receptor antagonist, reversibly inhibits monoamine oxidase A and B. Sci. Rep..

[60] Schrödinger Inc (2012). Maestro, Version 9.3.

[61] Acton A., Banck M., Bréfort J., Cruz M., Curtis D., Hassinen T., Heikkilä V., Hutchison G., Huuskonen J., Jensen J. (2011). Ghemical, Version 3.0.

[62] Clark M., Cramer R.D., Van Opdenbosch N. (1989). Validation of the general purpose Tripos 5.2 Force Field. J. Comput. Chem..

[63] Jones G., Willett P., Glen R.C., Leach A.R., Taylor R. (1997). Development and validation of a genetic algorithm for flexible docking. J. Mol. Biol..

