# Supplementary material for: Rivastigmine–Benzimidazole Hybrids as Promising Multitarget Metal-Modulating Compounds for Potential Treatment of Neurodegenerative Diseases
Source: Int J Mol Sci. 2023 May 5;24(9):8312. doi: 10.3390/ijms24098312 (PMC10179505; doi:10.3390/ijms24098312)
Supplement: Supplementary file 1 [file ijms-24-08312-s001.zip › ijms-2271295-supplementary.pdf]

International Journal of *Molecular Sciences*

Supplementary Materials

Rivastigmine–Benzimidazole Hybrids as Promising Multitarget Metal-Modulating Compounds for Potential Treatment of Neurodegenerative Diseases

David Vicente-Zurdo [1,2], Leonardo Brunetti [1,3], Luca Piemontese [3], Beatriz Guedes [4], Sandra M. Cardoso [4,5], Daniel Chavarria [6], Fernanda Borges [6], Yolanda Madrid [2], Sílvia Chaves [1,*] and M. Amélia Santos [1,*]

[1] Centro de Química Estrutural, Departamento de Engenharia Química, Institute of Molecular Sciences, Instituto Superior Técnico, Universidade de Lisboa, Av. Rovisco Pais 1, 1049-001 Lisboa, Portugal
[2] Department of Analytical Chemistry, Faculty of Chemistry, Complutense University of Madrid, Avenida Complutense s/n, 28040 Madrid, Spain
[3] Department of Pharmacy—Pharmaceutical Sciences, University of Bari Aldo Moro, via E. Orabona 4, 70125 Bari, Italy
[4] CNC—Center for Neuroscience and Cell Biology, University of Coimbra, 3000-370 Coimbra, Portugal
[5] FMUC—Faculty of Medicine, University of Coimbra, 3000-370 Coimbra, Portugal
[6] CIQUP-IMS, Department of Chemistry and Biochemistry, Faculty of Sciences, University of Porto, Rua do Campo Alegre s/n, 4169-007 Porto, Portugal
* Correspondence: silvia.chaves@tecnico.ulisboa.pt (S.C.); masantos@tecnico.ulisboa.pt (M.A.S.)

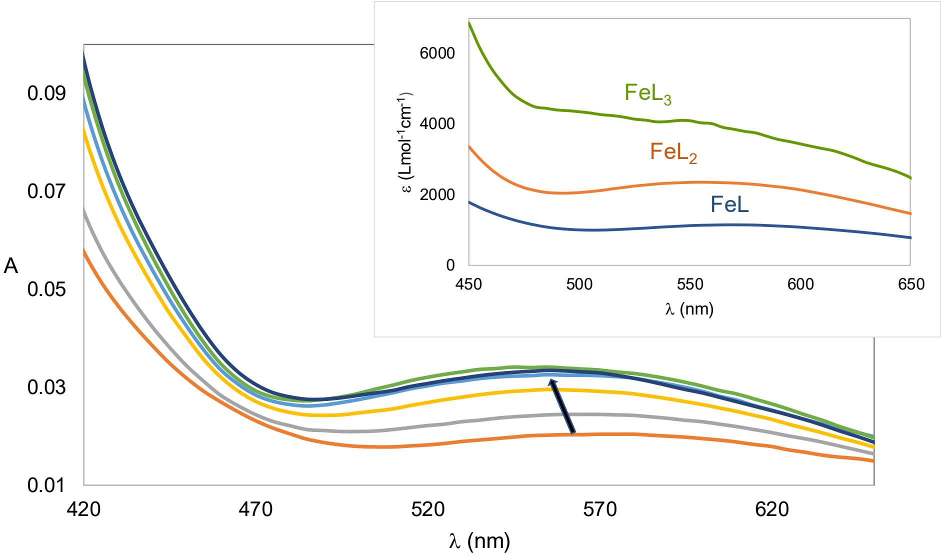

Figure S1. Absorption spectra for the Fe(III)/**5a** 1:3 system (2.71 < pH < 5.91) in 50% (w/w) DMSO/H_2O ($C_L = 4 \times 10^{-5}$ M) and outset with individual calculated spectra by Psequad.

a) b)

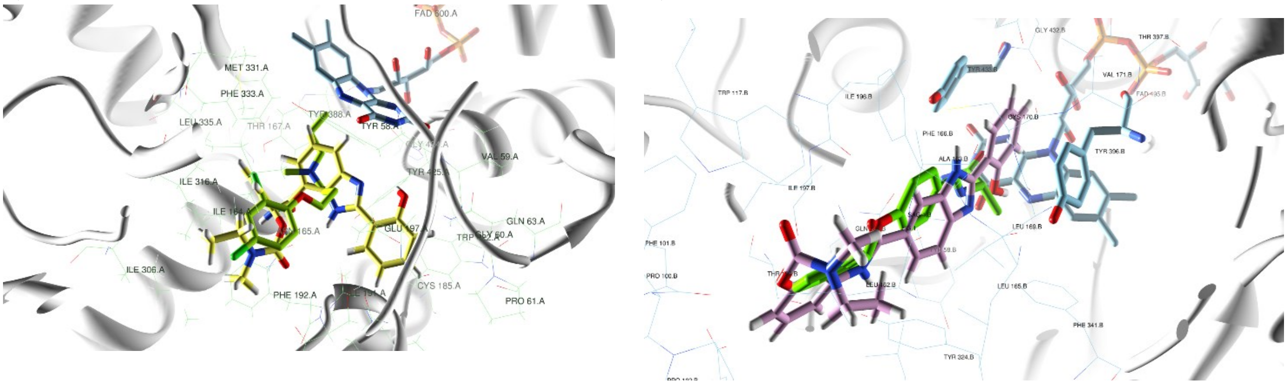

Figure S2. Docking results for RIV-BIM hybrids: (**a**) **5a** (yellow) with *h*-MAOA (PDB code: 2BXR) and (**b**) **4a** (pink) with *h*-MAOB (PDB code: 2V5Z). Pose comparisons with original ligand (green).
